# Gold/Pentablock Terpolymer Hybrid Multifunctional Nanocarriers for Controlled Delivery of Tamoxifen: Effect of Nanostructure on Release Kinetics

**DOI:** 10.3390/molecules27123764

**Published:** 2022-06-11

**Authors:** Maria-Teodora Popescu, Constantinos Tsitsilianis

**Affiliations:** Department of Chemical Engineering, University of Patras, 26500 Patras, Greece; theo_popi@yahoo.com

**Keywords:** pentablock, poly(ε-caprolactone), poly(2-vinylpyridine), poly(ethylene oxide), amphiphilic polymeric micelles, gold nanoparticles, organic/inorganic hybrid, Tamoxifen, drug delivery

## Abstract

Here, we describe the preparation and characterization of organic/inorganic hybrid polymer multifunctional nanocarriers. Novel nanocomposites of gold nanoparticles using pH-responsive coordination pentablock terpolymers of poly(ε-caprolactone)-*b*-poly(ethylene oxide)-*b*-poly(2-vinylpyridine)-*b*-poly(ethylene oxide)-*b*-poly(ε-caprolactone), bearing or not bearing partially quaternized vinylpyridine moieties, were studied. The template morphology of the coordination pentablock terpolymer at physiological pH ranges from crew-cut to multicompartmentalized micelles which can be tuned by chemical modification of the central block. Additionally, the presence of 2VP groups allows the coordination of gold ions, which can be reduced in situ to construct gold@polymer nanohybrids. Furthermore, the possibility of tuning the gold distribution in the micelles, through partial quaternization of the central P2VP block, was also investigated. Various morphological gold colloidal nanoparticles such as gold@core-corona nanoparticles and gold@core-gold@corona nanoparticles were synthesized on the corresponding template of the pentablock terpolymer, first by coordination with gold ions, followed by reduction with NaBH_4_. The pentablock and gold@pentablock nanoparticles could sparingly accommodate a water-soluble drug, Tamoxifen (TAX), in their hydrophobic micellar cores. The nanostructure of the nanocarrier remarkably affects the TAX delivery kinetics. Importantly, the hybrid gold@polymer nanoparticles showed prolonged release profiles for the guest molecule, relative to the corresponding bare amphiphilic pentablock polymeric micelles. These Gold@pentablock terpolymer hybrid nanoparticles could act as a multifunctional theranostic nanoplatform, integrating sustainable pH-controlled drug delivery, diagnostic function and photothermal therapy.

## 1. Introduction

Advances in nanobiotechnology have resulted in the development of novel materials for enhanced drug delivery and imaging applications, particularly in cancer research [1,2,3,4]. Despite extensive innovation over the past decade, there is still a need for integrated, easily adaptable drug delivery and imaging modalities, especially those for the delivery and monitoring of highly toxic compounds in vivo. Polymer nanoparticles are versatile materials for this purpose, due to their enhanced drug loading capacity, biological stability and extended in vivo circulation [5,6]. Remarkable progress has been made during the last few decades in the development and investigation of nanoparticles formed by supramolecular organization of amphiphilic block copolymers in aqueous systems [7,8,9,10,11]. The use of water as the solvent medium offers many advantages for potential applications in biological environments. In most cases, spherical micelles are formed in water, while different morphologies have also been reported, ranging from cylindrical and plate-like micelles to vesicles and complex micellar aggregates, when varying the copolymer structure, molecular weight and composition [12,13]. Progress in polymer synthesis has made it possible to prepare external-stimuli responsive block copolymers, known as “smart” block copolymers. These materials respond to changes in their environment such as pH, temperature and salt concentration and undergo micellization in aqueous media [14,15,16]. The fully reversible micellization process controlled by proper adjustment of the external stimuli are considerable advantages of these systems.

An interesting feature of block copolymer micelles is their potential use as nanoscopic reaction vessels to grow inorganic metal nanocrystals, resulting in hybrid nanocomposite materials [17,18,19]. The properties of these colloidal metal nanoparticles, which are determined by their particle size, shape and internal structure, are greatly different from those of their bulk counterparts and make them particularly attractive for applications in catalysis, optical devices and drug delivery. In such systems, the nonpolar block of the copolymer forms the corona, which provides stabilization, while the polar block forms the core, which can issolve metal compounds by coordination. Such micelles can be considered as nanoreactors or templates [20] where nucleation and growth of metal nanoparticles upon reduction are restricted to the mesoscale level and the size and morphology of the resulting metal colloidal nanoparticles depend on the size and morphology of the template micelles.

Of all the metal colloidal particles, gold nanoparticles (AuNPs) are particularly attractive due to their unique electronic and catalytic properties, and biocompatible nature [21,22,23,24]. Moreover, AuNPs are excellent photoabsorbers and have been extensively used in photothermal therapies in the form of nanosized spheres, rods and shells due to their very high photothermal conversion efficiencies. However, AuNPs combined with anticancer drugs integrated in hybrid polymeric formulations can be utilized as promising photochemotherapeutic agents [25,26]. Thus, a large number of polymer molecules have been selected to decorate the surface of gold nanoparticles for a variety of applications in the field of biotechnology [27,28,29,30,31]. The polymer chains grafted/coated on the surfaces of gold nanoparticles can not only intensively enhance the stability of gold cores, but can also functionalize the gold core due to the special properties of outside polymer layers. Particularly, “smart” nanocomposites consisting of gold nanoparticles and intelligent polymers display significant and remarkable aspects [32,33], and such a combination exploits a facile path for multifunctional materials and facilitates a variety of novel technological applications. For example, Au/polymer nanocomposites that respond to specific stimuli found in vivo (i.e., pH) should provide smart nanocarriers for tuning drug release. The stimuli sensitivity of the polymer template endues the smart gold nanocomposites with response reversibility and significant performances for intelligent materials. Subsequently, poly(ethylene glycol) (PEG) [34,35] can be readily conjugated onto the surface of AuNPs to improve biocompatibility, blood-circulation times and accumulation in solid tumors through enhanced permeability and retention (EPR) effects.

Thus, we report herein on the preparation of novel gold nanocomposites through coordination with pH-responsive amphiphilic pentablock terpolymers of ABCBA architecture. The polymers consisted of a pH-sensitive poly(2-vinylpyridine) (P2VP) central block, covalently bonded at both ends by hydrophilic poly(ethylene oxide) (PEO) blocks, end-capped with hydrophobic poly(ε-caprolactone) (PεCL) blocks. Moreover, the pH sensitive P2VP block can be readily partially quaternized, hence bearing permanently positively charged and hence hydrophilic moieties. In our previous works, morphological transitions from core–shell-corona micelles to crew-cut or multicompartmentalized micelles and to vesicles were observed for a PCL-PEO-P2VP-PEO-PCL pentablock copolymers in phosphate-buffered water by tuning pH, chemical modification of P2VP block and preparation strategy [36,37]. Therefore, this pentablock terpolymer offers various possibilities as template towards hybrid nanocarriers.

The affinity of pH sensitive P2VP for gold nanoparticles is well documented [38,39]. Composite Janus particles containing gold nanoparticles in P2VP-PEO block copolymers have been reported from dissociation and reorganization of polymer vesicles into Janus micelles [40]. Various morphological gold colloidal nanoparticles using a thermo-responsive and pH-responsive coordination triblock copolymer of PEO-P2VP-PNIPAM has been investigated. Discrete gold nanoparticles, gold@polymer core–shell nanoparticles, and gold nanoparticle clusters were synthesized by tuning pH and temperature conditions [41]. Gold-loaded PEO-*b*-PCL block copolymer micelles with a core–shell structure were prepared, and their stability in an aqueous system was dependent on the gold feed ratio [42]. The PCL-*b*-PEO-*b*-P2VP-*b*-PEO-*b*-PCL type architecture investigated herein should enhance the diversity and tunability of the self-assembled hybrid nanostructures. Additionally, partial quaternization of P2VP block should endow the nanoparticles with increased affinity for the AuCl_4_^−^ since quaternary ammonium cations are able to combine with the surface of the gold nanoparticles and act as a stabilizer using strong electrostatic attraction [43]. Herein, we follow the micelle transformations of these pentablock terpolymers after metalation with hydrochloroauric acid, followed by reduction. We describe the preparation and characterization of hybrid nanoparticles for use as multimodal carriers for drugs and imaging agents, on the pentablock terpolymer micellar template.

Furthermore, we attempted to explore the potential of such pentablock terpolymer hybrid assemblies as drug nanocarriers. Tamoxifen (TAX), an antiestrogen drug used to treat breast cancer, [44] was selected as model hydrophobic drug and loaded in the pentablock polymeric nanoparticles. The release kinetics was evaluated for the bare nanoparticles and for the gold@pentablock hybrid nanocomposites. The structure of the hybrids significantly controls the TAX delivery kinetics.

## 2. Materials and Methods

### 2.1. Materials

Tamoxifen (TAX, free base), hydrogen tetrachloroaurate (HAuCl_4_) and sodium borohydride (NaBH_4_) were purchased from Sigma-Aldrich (St. Louis, MO, USA). All solvents used had analytical or HPLC grade and were purchased from Sigma-Aldrich (St. Louis, MO, USA). The polymers used in this study were the pentablock terpolymer PCL_46_-PEO_199_-P2VP_598_-PEO_199_-PCL_46_ (P5b) and its partially quaternized analogue, PCL_46_-PEO_199_-P(2VP-*co*-2VP_q_)_598_-PEO_199_-PCL_46_ (Q5b). Detailed synthesis and characterization of these polymers have been described in a previous publication [37]. Their molecular characteristics are displayed in Table 1.

Ultrapure water was provided by means of an ELGA Medica-R7/15 (ELGA Labwater, Woodridge, IL, USA).

### 2.2. Preparation of Polymer Nanoparticles

P5b or Q5b terpolymer nanoparticles were prepared by addition of ultrapure water to the pentablock terpolymer solutions in a water-miscible organic solvent. Next, 10 mL of water were added dropwise to 50 mL of 2 mg/mL solution of pentablock terpolymer in *N,N*-dimethylformamide (DMF) (control micelles) or DMF containing 10 mg of TAX (TAX formulations), under vigorous stirring for 2 h. Then, the solutions were placed in dialysis membrane (MWCO 12,000–14,000 Da, Thermo Fisher, Hampton, NH, USA) and dialyzed against PBS pH 7.4 for several days.

### 2.3. Gold-Pentablock Micellar Nanoparticles

Gold-P5b or gold-Q5b terpolymer micellar nanoparticles were prepared first by adding a given volume of HAuCl_4_ aqueous solution to the as obtained P5b or Q5b terpolymer nanoparticles, respectively, by dialysis (blank control micelles, or TAX-loaded micelles), as described in Section 2.2. The concentration of the HAuCl_4_ aqueous solution was 0.10 mg/mL, and its pH was equal to that of the corresponding aqueous solution of the P5b or Q5b terpolymers. The molar ratio of 2VP units to Au^3+^ was 4:1. Reduction of Au^3+^ was then carried out by addition of an excess of aqueous NaBH_4_ solution, and the unreacted NaBH_4_ was eliminated by dialysis against PBS pH 7.4. The characteristic dark red color of AuNPs appeared and was stabilized after 1 h. For the pH-dependent measurements, the pH was adjusted by adding appropriate amounts of HCl 0.1 M to the final solutions.

### 2.4. TAX Encapsulation

The loading (%) of TAX in the P5b and Q5b nanoparticles was determined from the calibration curves of TAX 5–50 μg/mL in PBS pH 7.4 and DMF, by UV-vis absorbance at λ_max_ = 280 nm (U-2001 UV–VIS spectrophotometer, Hitachi, Schaumburg, IL, USA). The quantification of TAX loading in Au/polymer hybrid micelles is complicated by the overlap in absorption spectra of the two components in the UV-vis. For this reason, we have alternatively calculated it from the NaCN-induced decomposition experiment. First, 1 mL of an aqueous NaCN solution (0.1 M) was added to 1 mL of a solution of the Au/polymer nanoparticles in PBS, followed by agitation of the mixture until a colorless solution was obtained (48 h). The concentration of the guest compound was then calculated based on the Beer–Lambert law (ε = 1.1 × 10^4^ at 280 nm). Then, the micelles were diluted to DMF (90% *v*/*v*) to ensure full decomposition of the micelles and the TAX concentration was measured. The TAX loading (%) was calculated from Equation (1):

(1) $$Loading\;\left(\%\right)=\left(1-\frac{A_{before\;micelle\;disruption\;}\;}{A_{after\;micelle\;disruption}}\right)\times 100$$

where *A_before micelle disruption_*/*A_after micelle disruption_* = weight of unloaded TAX/weight of total TAX used.

### 2.5. Drug Release

The release of TAX was monitored by UV-vis spectroscopy under “sink” conditions (achieved by placing diluted polymer solutions in dialysis bags, MWCO: 12,000) against PBS pH 7.4. At specific time-points, samples were removed, and drug release was measured. The volume of the solution was kept constant by renewing the receiving medium with PBS pH 7.4 after each sampling. The cumulative drug release of TAX was calculated from the following Equation (2):

(2) $$Cumulative\;drug\;release\;\left(\%\right)=\left(\frac{W_{t}}{W_{0}}\right)\times 100$$

where *W_t_* is the weight of drug released at time *t*, and *W*_0_ is the total TAX loaded in the polymeric micelles.

### 2.6. Techniques

*Dynamic Light Scattering (DLS):* Autocorrelation functions *C*(*q*,*t*) were measured with a BI-9000AT/Turbocorr digital correlator from a light source of He-Ne laser (632.8 nm) (Brookhaven Instruments, Holtsville, NY, USA). Cumulant and CONTIN analysis were performed through BI-DLSW software (Brookhaven Instruments, Holtsville, NY, USA). *UV-vis Spectroscopy:* A U-2001 Hitachi spectrophotometer (Hitachi, Schaumburg, IL, USA) was used to obtain absorption spectra between 300 and 700 nm. Spectra were recorded in a 1 cm path length quartz cuvette at 25 °C with a resolution of ±2 nm. *Zeta Potential measurements* were carried out at 25 °C, using a Malvern Nano Zetasizer 5000 (Malvern, UK) equipped with an He–Ne laser at 633 nm. *TEM:* High Resolution Transmission Electron Microscopy (TEM) experiments were carried out on a JEM 2100 microscope (JEOL, Tokyo, Japan) operating at 200 kV. The copolymer solutions were placed on carbon grids, kept in contact for 2 min and blotted. Grids were then allowed to air-dry.

## 3. Results and Discussion

### 3.1. Characterization of Au@Pentablock Terpolymer Nanocomposites

Our approach to synthesize the gold colloidal nanoparticles employed the pH-responsive coordination pentablock terpolymer of PCL_46_-PEO_199_-P2VP_598_-PEO_199_-PCL_46_ (P5b) and its partially quaternized analogue PCL_46_-PEO_199_-P(2VP-*co*-2VP*_q_*)_598_-PEO_199_-PCL_46_ (Q5b) which exist as different morphologies by tuning pH values [45,46]. The pentablock terpolymer nanoparticles were prepared by dissolving the polymers in an organic nonselective solvent (DMF), followed by the dropwise addition of PBS buffer (pH 7.4). This procedure rendered the hydrophobic blocks (PCL and P2VP) insoluble, triggering the self-assembly into the “kinetically frozen” hydrophobic core [47]. Finally, DMF was dialyzed, and the micelles were recovered in PBS buffer (pH 7.4).

At pH 3.0, the P2VP segments were highly protonated, adopting a stretched conformation; therefore the driving force for the self-assembly mainly arose from the hydrophobic PCL and nonprotonated P2VP blocks. This morphology has been ascribed to a core–shell-corona type of micelles, with the PCL/P2VP block forming the hydrophobic core, surrounded by protonated P2VP and P(2VP-*co*-2VP_q_) shell and by hydrophilic PEO coronal chains. At physiological conditions, the pH-responsive coordination P2VP block is insoluble, and the pentablock terpolymer P5b self-assembles into crew-cut micelles with the hydrophobic PCL/P2VP blocks as a segregated core [48] and the hydrophilic PEO looping chains as the corona. At pH 7.4, the quaternized terpolymer Q5b forms spherical micelles with an inner hydrophobic PCL/P2VP core, caging 2VP_q_ hydrophilic charged moieties, surrounded by a 2VP_q_/PEO mixed corona.

**Scheme 1:**
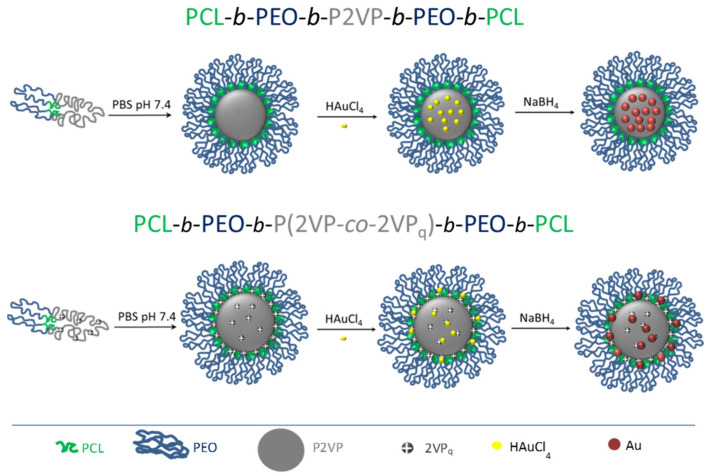
Synthesis of gold nanoparticle onto polymer micellar templates in PBS buffer at pH 7.4.

The Au^3+^ ions are first coordinated with nitrogen atoms from 2VP block of the pentablock terpolymer, at physiological pH, which exhibit micellar morphology as demonstrated above, and then reduced by NaBH_4_ to form gold@polymer nanoparticles. The micellization of the pentablock terpolymer and synthesis of the corresponding gold colloidal nanoparticles are shown in Scheme 1.

**Figure 1:**
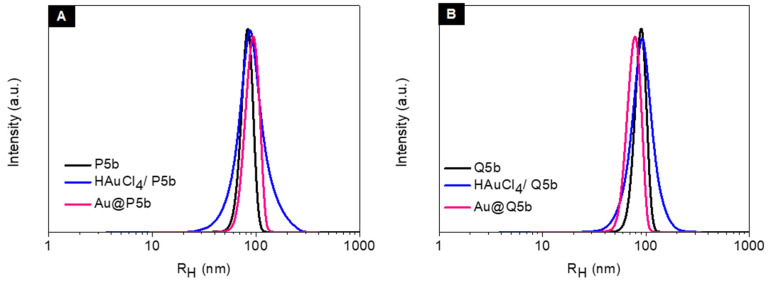
Size distribution of bare polymeric micelles (black), HAuCl_4_ doped micelles (blue) and Au@pentablock terpolymer nanoparticles (magenta) prepared with P5b (**A**) and Q5b (**B**) in pbs, pH 7.4.

Tetrachloroauric acid is selectively taken up by the pyridine units within the core of the pentablock terpolymer micelles. Incorporation of HAuCl_4_ results in local protonation of the 2VP units (protonation only of those units which participate in complexation), followed by electrostatic interaction of the quaternary ammonium species with AuCl_4_^−^ anions. When HAuCl_4_-filled micelles are reduced with sodium borohydride, two events take place: (i) gold nanoparticle formation and (ii) deprotonation of pyridine units. These have been monitored by DLS (Figure 1). Protonation might result in swelling of the micelle cores. However, since for the system herein, the coordination has taken place at physiological pH, incorporation of HAuCl_4_ (at molar ratios of 4VP: Au of 4:1) in the as-prepared pentablock terpolymer (P5b) micelles does not result in a significant increase in the micelle size (Figure 1A). The hydrodynamic radius of the micelles loaded with HAuCl_4_ is 86 nm. After reduction, *R*_H_ is measured 95 nm for the micelles containing gold nanoparticles. The increase in size could be the result of incorporation of the metal in the micellar core. On the contrary, the hybrid micelles from the partially quaternized pentablock terpolymer analogue (Q5b) seem to adopt a slightly more compacted conformation, as observed in Figure 1B, probably due to the suppression of intramolecular repulsive interactions of charged 2VP_q_ moieties upon gold reduction.

**Figure 2:**
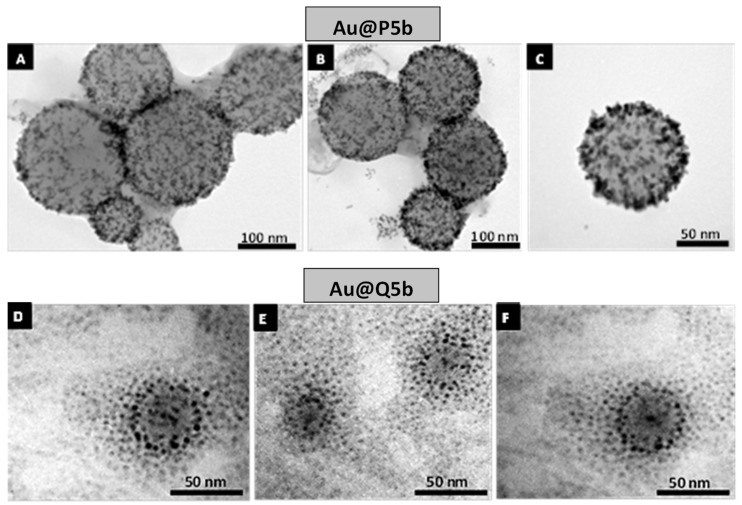
TEM images of gold nanoparticles coated with P5b (**A**–**C**) and Q5b (**D**–**F**) in PBS at pH 7.4.

At pH 7.4, the P2VP block is hydrophobic; thus, the P5b pentablock copolymer self-assembles into crew-cut micelles with the hydrophobic PCL/P2VP blocks as the core and the hydrophilic PEO blocks as the corona. The ions of Au^3+^ can first be loaded into the P2VP core of the micelles and then are reduced into gold nanoparticles to form Au@P5b crew-cut nanoparticles. The TEM image of the Au@P5b nanoparticles is shown in Figure 2A–C. The average diameter of the Au@P5b nanoparticles is about 110 nm. The TEM micrographs of these samples display well-defined particles, with about a 26% increase in size relative to the bare polymeric nanoparticles. Small gold nanoparticles are observed to be densely deposited in the micelles. The hybrid micelles of P5b again seem to be interconnected with some agglomeration of gold nanoparticles at the edges. Typically, NaBH_4_ provides a fast nucleation, and the growth of metal particles is controlled by the polymeric ligand. It is presumed that the larger particles are formed on the borders of micelle cores due to exchange between micelles. Previously, it has been shown that for a P2VP-*b*-PEO diblock copolymer, the chloroauric acid is completely bound to the micelles, thus the smaller AuNPs form inside the micelle cores, because of nucleation, while larger AuNPs are the result of the exchange between micelles due to collision [49]. The reduction behavior of gold ions is dependent on the pH of the solution during reduction treatment. Because the pH of the aqueous polymer solution was 7.4, the ion-doped nanoparticles were in a shrunken state (the PCL/P2VP were hydrophobic but water dispersible because of the PEG corona). Therefore, the NaBH_4_ molecules penetrated the micelles only to a small extent, causing the reduction of the gold ions mainly at the core/corona interface.

The quaternized pentablock terpolymer exists as spherical micelles at pH 7.4 with 2VP_q_ moieties mostly present at the outer corona, while some are caged within the hydrophobic P2VP/PCL core. Metal binding is due to electrostatic attraction and hydrogen bonding of protonated 2VP units with AuCl_4_^−^. When the ions of AuCl_4_^−^ are added into the polymer solution, they first coordinate with nitrogen atoms of the P(2VP-co-2VP_q_) block of the terpolymer and then are reduced into gold nanoparticles. Moreover, the strong polyelectrolyte nature of 2VP_q_ moieties would result in intimate association of the AuCl_4_^−^ counterions prior to the *in situ* reduction to zero-valent gold [43]. This should ensure efficient adsorption of the polymer chains onto the gold surface. Figure 2D–F show the TEM image of the resultant gold nanoparticles. The average diameter of the Au@Q5b nanoparticles is about 90 nm, as measured from the TEM micrographs, exhibiting a thin dark ring outside the gray core. These must be the AuNPs, located within the 2VP_q_ shell and still surrounded by the PEO corona. They are densely located within the 2VP_q_ shell and are less packed with growing distance.

The gold nanoparticles are present at the outer layer of the micelles, and some are visible in the inner micellar compartment. This could be due to selective reduction of the gold ions mainly at the micelle surface, where the 2VP_q_ would be located. After reduction, gold nanoparticles are formed at the PEO/P2VP interface and retain their swelled structure. We had assumed in a previous study that the quaternized pentablock terpolymer micelles formed at physiological pH are spherical micelles composed of PCL/P2VP core caging 2VP_q_ cationic moieties [37]. Herein, the distribution of gold nanoparticles into the inner core and the outer corona confirms this hypothesis.

This surprising difference in the deposition of gold nanoparticles in the P2VP core for the P5b and inner/outer compartments for Q5b is likely due to different packing in the as-prepared micelles filled with HAuCl_4_. One can assume that, in the former case, the P2VP chains are very densely packed within the micelle core, obstructing diffusion of the ions, thus leading to the formation of gold@core-corona type nanoparticles. In the case of the quaternized pentablock terpolymer, the micellar hydrophobic compartments are looser due to the presence of repulsive interactions of the charged 2VP_q_ moieties enhancing diffusion and, thus, distribution of gold nanoparticles in the inner compartments of the micelles. The template micelles formed by the partially quaternized pentablock terpolymer Q5b lead to the formation of gold@core-gold@corona hybrid nanoparticles.

**Figure 3:**
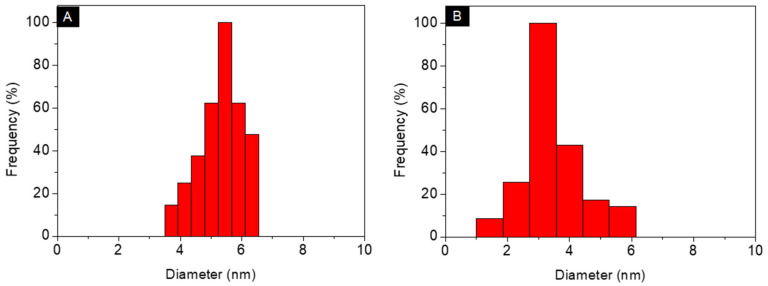
Particle size (diameter) histograms of the gold nanoparticles prepared with P5b (**A**) and Q5b (**B**) derived from TEM images presented in Figure 2.

The diameter of the gold nanoparticles within the polymer micelles was independently determined from the TEM images using ImageJ software for analysis. The number of individual nanoparticles in each TEM images is more than 50 units and is sufficient to calculate the particle diameter reliably. The analysis result was reproducible and was consistent over the different areas. Figure 3 shows histograms of size distribution calculated from the TEM images. As clearly observed, the gold nanoparticles are spherical with an average particle diameter corresponding to 5.2 nm for P5b (Figure 3A) and 3.6 for Q5b (Figure 3B), respectively. The histograms show that Q5b hybrid micelle possesses a broader size distribution of gold nanoparticles, which implies less stringent confined conditions for nanoparticle formation.

The size of the nanoparticles prepared by the reduction of the gold species in pentablock terpolymer micelles normally depends on several parameters, such as the type of reducing agent and the loading of the metal precursor [50]. The former parameter determines the rate of nucleation and particle growth: slow reduction produces large particles, while fast reduction gives small particles. In this work we used only NaBH_4_ (a mild reducing agent) at constant pH of the reacting solution, thus maintaining the rate of nucleation for both polymeric systems.

The metal loading factor determines the local concentration of metal species: the higher the concentration, the larger the particles [50]. Accordingly, multiple vinylpyridine groups participate in the reduction of a single HAuCl_4_ molecule, as the full reduction of Au^3+^ to Au^0^ requires three electron transfers [51]. Assuming each pyridine donates only one electron (reacts only once), and that three pyridine units are required to fully reduce one molecule of HAuCl_4_, then there are theoretically enough amine groups available to reduce all of the Au^3+^ for the loading ratio of 4:1 used herein. To contribute to the stabilization of the AuNPs, the 2VP_q_ cation chelates with the metal surface through its midpoint nitrogen atom, further protecting the AuNPs from aggregation [43]. This binding results in the observed decrease in gold particle size when dispersed within the partially quaternized pentablock terpolymer Q5b micellar template.

Another important factor is the core segment density; specifically, a higher core-density will provide increase diffusion limitations [49,52]. Difficult diffusion of metal species in HAuCl_4_-filled P5b micelles, in addition to PEO bridging between adjacent micelles, result in the facilitated exchange between micelles, leading to uneven distribution of gold nanoparticles.

**Figure 4:**
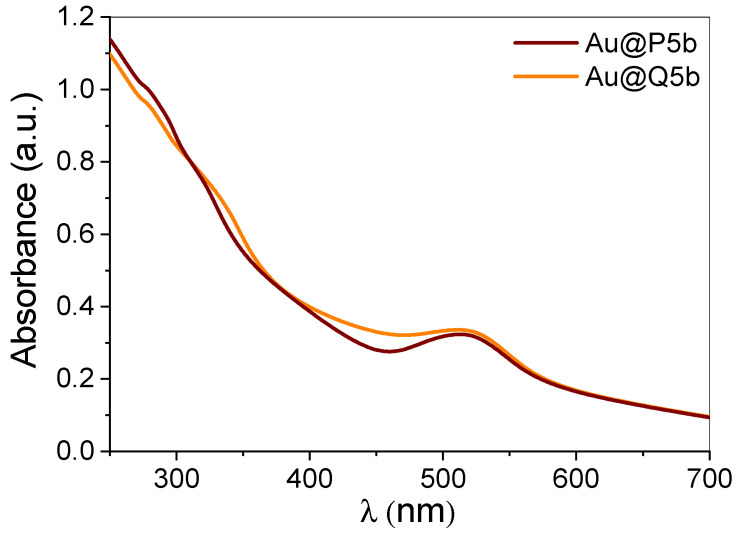
UV-vis spectra of gold@pentablock terpolymer hybrid nanoparticle aqueous solution of 0.2 mg/mL in PBS at pH 7.4.

It is well documented that UV-vis spectroscopy can be used to determine the aggregation state of gold colloids [53,54]. For example, highly dispersed gold particles with diameters of ~20 nm exhibit an absorbance peak of approximately 520 nm. As the gold particle size decreases to about 5–10 nm, the extinction peak shifts to around 515 nm. Figure 4 presents UV-vis spectra of the micellar solutions P5b and Q5b after gold nanoparticle formation at physiological pH. The positions of the absorption peaks with maximum absorption at 518 nm for P5b and 516 nm for Q5b, respectively, and their widths are very close for the two samples, independent of the particle size. This is in good agreement with the prediction of a classical Mie theory for spherical particles with diameters of about 10 nm and smaller, where the plasmon band position becomes independent of the particle size [55].

The results of UV-vis absorption spectral measurements are consistent with that of the TEM observations. Furthermore, it is found that the gold colloids are very stable, and no precipitation can be detected when the sample has been kept for six months at room temperature.

### 3.2. Release of TAX from Hybrid Nanoparticles at Physiological pH

The amphiphilic terpolymers studied herein present good biocompatibility, as previously proven by Van Butsele et al. for a linear triblock copolymer based on the same type of building blocks [45]. Thus, the potential of the pentablock nanoparticles for drug-delivery applications has been further explored by encapsulation of a poorly water-soluble drug within the pentablock terpolymer bare or hybrid nanoparticles. Tamoxifen (TAX), an antiestrogen drug used to treat breast cancer, was selected as the model hydrophobic drug and this drug-loaded carrier system was evaluated ex vivo [44]. The drug-loading capacities of these micelles and their drug-release profiles were studied under physiologically relevant conditions.

**Figure 5:**
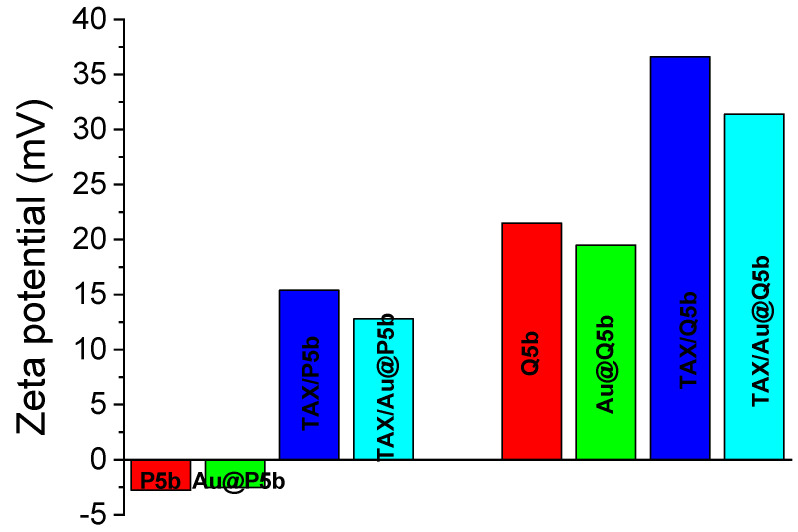
Zeta potential values of pentablock terpolymer bare and hybrid nanoparticles before and after encapsulation of TAX.

The nanoparticle-payload conjugates were prepared by a solvent displacement method. First, the pentablock terpolymers and guest were dissolved in DMF followed by dialysis against buffer. In the gradual replacement of the organic solvent by aqueous medium during dialysis, self-assembly of hydrophobic blocks provides the driving force for the micelle formation and the drug loading in the micelle cores. Therefore, the interactions of drugs with both the hydrophobic block and solvent determine the drug-loading amount [46]. The reduction of TAX-loaded Au@polymer micelles was performed as described above for the empty polymer nanoparticles. At physiological pH, the pentablock terpolymer nanoparticles, with or without gold nanoparticles, showed a surface charge that was negative for P5b and positive for Q5b, respectively (Figure 5). However, drug encapsulation switched the surface charge to predominantly positive for both systems due to the presence of surface-localized drugs [56]. Moreover, no change in the hydrodynamic diameters of drug-loaded polymeric micelles was observed by DLS (not shown) implying that the micelles have preserved their integrity during drug loading.

Thus, the increased zeta potentials of the drug-loaded micelles suggest that drug molecules may be solubilized not only within the micelle cores but also close to the micelle periphery. Cationic surface charge is desirable, as it promotes interaction of the nanoparticles with cells, and, hence, increases the rate and extent their internalization [57].

**Figure 6:**
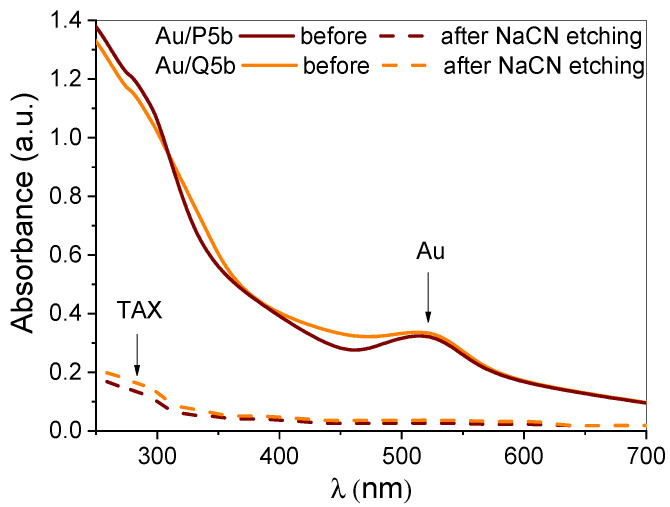
UV-vis spectra of gold@pentablock terpolymer nanoparticles loading TAX, at 0.2 mg/mL in PBS at pH 7.4, before and after NaCN etching.

The encapsulation of TAX in the pentablock terpolymer hybrid micelles was obtained both in the presence and in the absence of gold nanoparticles. The amount of TAX loaded in different types of polymer particles at physiological conditions was determined by UV-vis spectroscopy at λ_max_ = 280 nm, and the loading was calculated based on the amount of guest molecule measured before and after micelle disruption. Micellar disruption was performed by two methods: solubilization in DMF or at acidic pH. It should be noted that at pH 3, corresponding to protonation of P2VP core, the absorbance of TAX was lower relative to that measured in organic solvent (DMF) for both pentablock terpolymers, illustrating entrapment of the hydrophobic drug within the P2VP/PCL core. Apparently, TAX is preferentially distributed within the P2VP core (>90% of total amount encapsulated), a fact that leads to the increased positive zeta potential observed. The quantification of TAX loading in the pentablock hybrid micelles is complicated by the overlap in absorption spectra of the two components in the UV region, where solutions of TAX exhibit an absorbance maximum at 280 nm. For the gold nanoparticle hybrids, the encapsulation of the entrapped guest molecule was determined from NaCN-induced decomposition experiments [58] (Figure 6). After etching, the plasmon absorption band of AuNP at 520 nm was completely decayed, while absorbance of guest compound remained.

**Figure 7:**
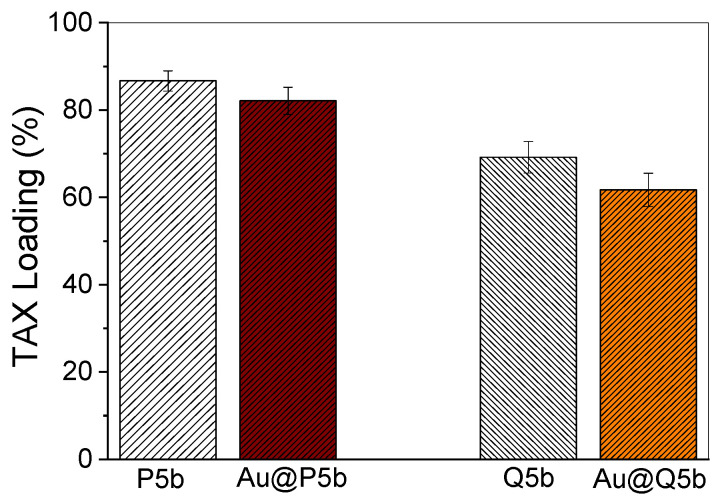
TAX loading (%) in pentablock terpolymer bare and hybrid micelles, in PBS at pH 7.4. Each point is the mean from three independent measurements and bars represent standard deviation of means.

The loading of TAX was quite high (>80%) when the formulations were prepared from the P5b pentablock copolymer, and significantly lower (>60%) for those prepared by Q5b pentablock terpolymer bearing quaternized moieties (Figure 7). This encapsulation is much higher than that previously reported for TAX loading within core shell micelles using a solvent displacement method. It is known that physical entrapment of hydrophobic drugs in block copolymer micelles is driven by drug solubilization within the hydrophobic micelle cores [59,60]; thus, it is reasonable that the drug will require a longer hydrophobic P2VP block to become entrapped. As a result, micelles with increased P2VP hydrophobic content showed more effective incorporation ability of TAX molecules because of increased hydrophobic interactions between the drug and the hydrophobic blocks.

Additionally, the loading percentage was slightly lowered for the gold nanoparticle hybrid micelles, probably because of P2VP core swelling by HAuCl_4_, resulting in escape of some TAX molecules. However, the extent of it is quite reduced with about ~10% lower encapsulation. The method of nanoparticle preparation and drug encapsulation is highly favorable for hydrophobic compounds, such as TAX, resulting in excellent entrapment levels (i.e., loading capacity: more than 6% of loading TAX per micelle weight) for both the pentablock terpolymers and their gold nanoparticle hybrids, which is a desirable parameter for increasing the efficiency of drug delivery. Note that in each case, the water solubility of TAX loaded micelles was up to 2.1 mg/mL, which is almost three orders of magnitude greater than the aqueous solubility of the corresponding free drug (the water solubility of tamoxifen is 50 μg/mL) [61]. TAX is an effective anticancer agent against breast cancer, but its clinical applications have been hindered by its extremely low solubility. By encapsulating TAX within the pentablock terpolymer micelles, water solubility of TAX is increased significantly.

The in vitro release behavior of TAX-loaded pentablock terpolymers and TAX-loaded Au@pentablock terpolymers hybrid micelles in PBS buffer solutions (pH 7.4) was studied. Because of the low solubility of TAX in aqueous media, the TAX-loaded micelles are dialyzed against a large volume of PBS to ensure that the drug progressively released from micelles is below its solubility limit, without precipitation, as reported previously [61]. This experimental setup is meant to mimic in vivo sink conditions, resulting in diffusional drug release from micelles.

**Figure 8:**
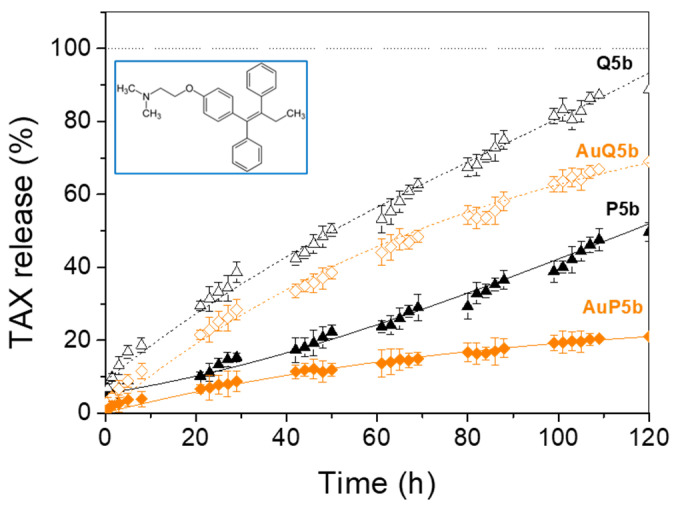
Cumulative amount of TAX (inset) released per time (hours) from pentablock terpolymer bare and hybrid micelles in PBS at pH 7.4. Each point is the mean from three independent measurements and bars represent standard deviation of means. The dotted lines are meant to provide guidance to the reader.

Figure 8 illustrates drug release from P5b and Q5b micelles and Au@P5b and Au@Q5b hybrid micelles, respectively. It is found that release of TAX from the micelles proceeds in two stages, with a relative rapid release observed in initial 10 h, followed by a slower sustained release. Both bare pentablock terpolymer micellar systems exhibit initial fast release of 8% for P5b and 19% for Q5b, respectively. The initial rapid release of TAX is probably attributed to the drugs adsorbed on the core/corona interface of micelles. After a fast release at first stage, the release rate of TAX slows down and becomes steady in a controlled manner. The drug release increases as a function of time in both systems but, micelles formed by P5b release a lower drug amount than micelles of Q5b.

In fact, after 120 h, 49% of the initially loaded drug was detected in the solution for pentablock terpolymer nanoparticles, vs. about 89% for partially quaternized pentablock terpolymer micelles. The faster release of TAX (about 81%) is possible due to the protonation of the amino group of TAX (pK_a_ of TAX is 8.85) and the expulsion by repulsive interaction with the quaternized 2VP moieties that enables faster diffusion of the encapsulated guest molecule.

For Au@polymer micelles, the drug release is suppressed due to the adsorption of Au onto the P2VP core, which hinders the diffusion of TAX to the external solution. The release rate of TAX for Au/micellar nanocariers was slower than that of bare micelles for the two different polymers, i.e., 76% and 43% reduction for Q5b and P5b, respectively. Moreover, regarding the hybrid nanocarriers, TAX-loaded micelles of Au@P5b released a significantly lower (only 30%) drug amount than that of Au@Q5b. The combination of gold and polymer with TAX results in a more sustained formulation compared to the bare micelles, since such a complex can release the drug at slower rate. Furthermore, the distribution of AuNPs within the micelles also seems to affect drug release. Specifically, the distribution of AuNPs within the core and the core–corona interface for the Au@Q5b formulation, results in faster escape of the encapsulate, while the presence of AuNPs predominantly in the core, as in the case of Au@P5b, leads to prolonged drug release. Release of physically encapsulated TAX from the micelle core was likely to proceed by diffusion, and the release rate was controlled by drug diffusion from the micelle core (i.e., the concentration of TAX in the aqueous release medium was maintained below the solubility limit in PBS) [61]. Similar results have been obtained by thermosensitive “shell-in-shell” nanospheres composed of poly(L,L-lactide-*co*-ethylene glycol) (PLLA-PEG) and poly(*N*-isopropylacrylamide-*co*-D,D-lactide) (PNIPAAm-PDLA) with a gold layer (Au@PLLA-PEG@PNIPAAm-PDLA) encapsulating BSA [62]. However, in that case, the protein molecule is inherently large, and retarded diffusion was expected. This sustained release is significantly improved with respect to other bare or composite micellar systems. For example, TAX diffusion from PCL nanoparticles was completed in less than 24h with a significant burst profile [63]. In the same study, when the nanoparticles were prepared in the presence of Pluronic F-68, TAX release was prolonged almost three-fold. The release of TAX from micelles formed by folic acid-poly(2-(methacryloyloxy) ethylphosphorylcholine)-*b*-poly(2-(diisopropylamino) ethyl methacrylate) (PMPC-PDPA) with hydrophobic pH sensitive PDPA block was attained in about 30 h. [64]. DOX release from folate-modified, polyethylene glycol-functionalized gold nanoparticles achieved completion within 50 h. [65]. Furthermore, release of TAX from poly(ethylene glycol) fumarate iron oxide composite nanoparticles attained 70% after 20 h. [66]. These comparisons also demonstrate that the pentablock terpolymer architecture, whether or not it bears 2VP_q_ moieties, provides improved stability of the micellar nanocarrier and enables a reduction in the drug release. Furthermore, gold@pentablock terpolymer hybrid nanoparticles offer additional sustainability and control over the drug-release kinetics and can be applicable as a molecular imaging drug delivery system.

Although we have not explored this issue in the present work, it is obvious that pH is an important stimulus that should affect the TAX release kinetics, since the P2VP central block is a weak cationic polyelectrolyte with a pK_a_ = 5. As we have shown in our previous papers with the same polymer [36,37] the reversible ionization of the hydrophobic P2VP induced by lowering pH accelerates the release of payloads due to the enhanced permeability of the P2VP-based hydrophobic parts of the micellar nanocarriers [37]. There is no reason to predict the opposite behavior for the TAX release through the cores of the micelles of this work, whether they are prepared from the quaternized version (only 19 mol% permanent ionization of P2VP) or not. Thus, in a tumor environment where pH is lower than 7.4, we expect faster release of TAX.

Another possibility of the AuNPs is that once they are exposed to light irradiation (laser monochromatic green light at 521 nm in our case), they can harvest light energy and convert it into heat, thereby raising the temperature of the surrounding environment (hyperthermia) to trigger tumor cell apoptosis known as photothermal therapy [67].

## 4. Conclusions

Herein, we reported the development of novel hybrid polymer nanoparticles encapsulating hydrophobic molecules as complex carriers for drugs and imaging agents. Nanocomposites of gold nanoparticles (AuNPs) using pH-responsive coordination biocompatible pentablock terpolymers of poly(ε-caprolactone)-*b*-poly(ethylene oxide)-*b*-poly(2-vinylpyridine)-*b*-poly(ethylene oxide)*-b*-poly(ε-caprolactone) were studied. The template morphology of the coordination pentablock terpolymer, at physiological pH, can be tuned by partial quaternization of the 2VP moieties, which will affect the structure of the micellar nanocarrier, namely distribution of AuNPs and/or degree of ionization of the core. The latter can be increased by lowering pH (reversible ionization). Various morphological gold colloidal nanoparticles, depending on the degree of quaternization of P2VP central blocks, such as gold@core-corona nanoparticles and gold@core-gold@corona nanoparticles, were prepared. The sparingly water-soluble drug, TAX, was used to evaluate the drug delivery capability of the nanocarriers. TAX is encapsulated within the hydrophobic micellar cores with remarkable loading capacity (higher than 6% of micelle weight). Importantly, the solubility of TAX within the micelles was almost three orders of magnitude greater than the aqueous solubility of the corresponding free drug. The *in vitro* release of guest molecule from the pentablock terpolymer micelles at physiological pH was controlled and extended for periods longer than 5 days. The micellar structure controlled the release kinetics. Faster drug release was achieved from micelles bearing cationic moieties (partially quaternized 2VP). More importantly, the incorporation of AuNPs in the micelles showed prolonged release profiles for the guest molecule relative to the corresponding bare amphiphilic pentablock micelles, which is also affected by the AuNP distribution and the degree of ionization (quaternization) of the P2VP cores. The latter can be further controlled by pH (reversible ionization). Provided that the doping of the TAX-loaded micellar nanoparticles with AuNPs allows us to trigger photothermal-induced tumor cell necrosis (photochemotherapy), these Gold@pentablock terpolymer hybrid nanoparticles could act as a multifunctional theranostic nanoplatform, integrating sustainable pH-controlled drug delivery, diagnostic function and photothermal therapy.

## Figures and Tables

**Table 1:** Molecular characteristics of the terpolymers.

POLYMER	M_w_	PDI	PEO wt%	P2VP wt%	QP2VP		PCL wt%
					wt%	mol%	
PCL_46_-PEO_199_-P2VP_598_-PEO_199_-PCL_46_ (P5b)	90,950	1.14	19.4	69	-	-	11.6
PCL_46_-PEO_199_-P(2VP-*co*-2VP*_q_*)_598_-PEO_199_-PCL_46_ (Q5b)	108,840	1.14	16	45.5	29.9	19	8.6

## Data Availability

The data presented in this study are available on request from the corresponding author.

## References

[1] Zhang X.Q., Xu X., Bertrand N., Pridgen E., Swami A., Farokhzad O.C. (2012). Interactions of nanomaterials and biological systems: Implications to personalized nanomedicine. Adv. Drug Deliv. Rev..

[2] Beija M., Salvayre R., de Viguerie N.L., Marty J.-D. (2012). Colloidal systems for drug delivery: From design to therapy. Trends Biotechnol..

[3] Mendes M., Sousa J.J., Pais A., Vitorino C. (2018). Targeted Theranostic Nanoparticles for Brain Tumor Treatment. Pharmaceutics.

[4] Klochkov S.G., Neganova M.E., Nikolenko V.N., Chen K., Somasundaram S.G., Kirkland C.E., Aliev G. (2021). Implications of nanotechnology for the treatment of cancer: Recent advances. Semin. Cancer Biol..

[5] Xiong X.-B., Binkhathlan Z., Molavi O., Lavasanifar A. (2012). Amphiphilic block co-polymers: Preparation and application in nanodrug and gene delivery. Acta Biomater..

[6] Kwon G.S., Kataoka K. (1995). Block copolymer micelles as long-circulating drug vehicles. Adv. Drug Deliv. Rev..

[7] Yu L., Wang H., Wang H.H., Urban V.S., Littrell K.C., Thiyagarajan P. (2000). Syntheses of Amphiphilic Diblock Copolymers Containing a Conjugated Block and Their Self-Assembling Properties. J. Am. Chem. Soc..

[8] Sarkar B., Ravi V., Alexandridis P. (2013). Micellization of amphiphilic block copolymers in binary and ternary solvent mixtures. J. Colloid Interface. Sci..

[9] Mohammadi M., Ramezani M., Abnous K., Alibolandi M. (2017). Biocompatible polymersomes-based cancer theranostics: Towards multifunctional nanomedicine. Int. J. Pharm..

[10] Kennemur J.G. (2019). Poly(vinylpyridine) Segments in Block Copolymers: Synthesis, Self-Assembly, and Versatility. Macromolecules.

[11] Nghiem T.-L., Chakroun R., Janoszka N., Chen C., Klein K., Wong C.K., Gröschel A.H. (2020). pH-Controlled Hierarchical Assembly/Disassembly of Multicompartment Micelles in Water. Macromol. Rapid Commun..

[12] Zhang L.F., Eisenberg A. (1995). Multiple Morphologies of "Crew-Cut" Aggregates of Polystyrene-*b*-poly(acrylic acid) Block Copolymers. Science.

[13] Liu Y., Liu B., Nie Z. (2015). Concurrent self-assembly of amphiphiles into nanoarchitectures with increasing complexity. Nano Today.

[14] Popescu M.-T., Tsitsilianis C., Papadakis C.M., Adelsberger J., Balog S., Busch P., Hadjiantoniou N.A., Patrickios C.S. (2012). Stimuli-responsive Amphiphilic Polyelectrolyte Heptablock Copolymer Physical Hydrogels: An Unusual pH-response. Macromolecules.

[15] Koonar I., Zhou C., Hillmyer M.A., Lodge T.P., Siegel R.A. (2012). ABC Triblock Terpolymers Exhibiting Both Temperature- and pH-Sensitive Micellar Aggregation and Gelation in Aqueous Solution. Langmuir.

[16] Pioge S., Fustin C.A., Gohy J.F. (2012). Temperature-Responsive Aqueous Micelles From Terpyridine End-Capped Poly(*N*-Isopropylacrylamide)-*Block*-Polystyrene Diblock Copolymers. Macromol. Rapid Comm..

[17] Bronstein L.M., Kramer E., Berton B., Burger C., Foster S., Antonietti M. (1999). Successive Use of Amphiphilic Block Copolymers as Nanoreactors and Templates:  Preparation of Porous Silica with Metal Nanoparticles. Chem. Mater..

[18] Mossmer S., Spatz J.P., Moller M., Aberle T., Schmidt J., Burchard W. (2000). Solution Behavior of Poly(styrene)-block-poly(2-vinylpyridine) Micelles Containing Gold Nanoparticles. Macromolecules.

[19] Mai Y., Eisenberg A. (2010). Controlled Incorporation of Particles into the Central Portion of Vesicle Walls. J. Am. Chem. Soc..

[20] Vriezema D.M., Aragones M.C., Elemans J.A.A.W., Cornelissen J.J.L.M., Rowan A.E., Nolte R.J.M. (2005). Self-Assembled Nanoreactors. Chem. Rev..

[21] Thakor A.S., Jokerst J., Zavaleta C., Massoud T.F., Gambhir S.S. (2011). Gold nanoparticles: A revival in precious metal administration to patients. Nano Lett..

[22] Tiwari P.M., Vig K., Dennis V.A., Singh S.R. (2011). Functionalized Gold Nanoparticles and Their Biomedical Applications. Nanomaterials.

[23] Saha K.S., Agasti S., Kim C., Li X., Rotello V.M. (2012). Gold nanoparticles in chemical and biological sensing. Chem. Rev..

[24] Gao J., Bender C.M., Murphy C.J. (2003). Dependence of the Gold Nanorod Aspect Ratio on the Nature of the Directing Surfactant in Aqueous Solution. Langmuir.

[25] Sun T., Wang Y., Wang Y., Xu J., Zhao X., Vangveravong S., Mach R.H., Xia Y. (2014). Using SV119-Gold Nanocage Conjugates to Eradicate Cancer Stem Cells Through a Combination of Photothermal and Chemo Therapies. Adv. Healthc. Mater..

[26] Joubert F., Pasparakis G. (2018). Hierarchically designed hybrid nanoparticles for combinational photochemotherapy against a pancreatic cancer cell line. J. Mater. Chem. B.

[27] Gibson M.I., Paripovic D., Klok H.-A. (2010). Size-Dependent LCST Transitions of Polymer-Coated Gold Nanoparticles: Cooperative Aggregation and Surface Assembly. Adv. Mater..

[28] Du B., Zhao B., Tao P., Yin K., Lei P., Wang Q. (2008). Amphiphilic multiblock copolymer stabilized Au nanoparticles. Colloids Surf. A.

[29] Kang Y., Taton T.A. (2005). Core/shell gold nanoparticles by self-assembly and crosslinking of micellar, block-copolymer shells. Angew. Chem. Int. Ed..

[30] Bae K.H., Choi S.H., Park S.Y., Lee Y., Park T.G. (2006). Thermosensitive Pluronic Micelles Stabilized by Shell Cross-Linking with Gold Nanoparticles. Langmuir.

[31] Siddique S., Chow J.C.L. (2020). Gold Nanoparticles for Drug Delivery and Cancer Therapy. Appl. Sci..

[32] Nash M.A., Lai J.J., Hoffman A.S., Yager P., Stayton P.S. (2010). “Smart” diblock copolymers as templates for magnetic-core gold-shell nanoparticle synthesis. Nano Lett..

[33] Dai Y., Zhang X. (2018). Recent Advances in Amphiphilic Polymers as the Stabilizers of Colloidal Gold Nanoparticles. Macromol. Mater. Eng..

[34] Asadishad B., Vossoughi M., Alemzadeh I. (2010). Folate-receptor-targeted delivery of doxorubicin using polyethylene glycol-functionalized gold nanoparticles. Ind. Eng. Chem. Res..

[35] Qian W., Murakami M., Ichikawa Y., Che Y. (2011). Highly efficient and controllable PEGylation of gold nanoparticles prepared by femtosecond laser ablation in water. J. Phys. Chem. C.

[36] Popescu M.-T., Tsitsilianis C. (2013). Controlled Delivery of Functionalized Gold Nanoparticles by pH-Sensitive Polymersomes. ACS Macro Lett..

[37] Popescu M.-T., Korogiannaki K., Marikou K., Tsitsilianis C. (2014). CBABC terpolymer-based nanostructured vesicles with tunable membrane permeability as potential hydrophilic drug nanocarriers. Polymer.

[38] Li D.X., He Q., Cui Y., Li J.B. (2007). Fabrication of pH-responsive nanocomposites of gold nanoparticles/poly(4-vinylpyridine). Chem. Mater..

[39] Suntivich R., Choi I., Gupta M.K., Tsitsilianis C., Tsukruk V.V. (2011). Gold nanoparticles grown on star-shaped block copolymer monolayers. Langmuir.

[40] Li X., Yang H., Xu L., Fu X., Guo H., Zhang X. (2010). Janus Micelle Formation Induced by Protonation/Deprotonation of Poly(2-vinylpyridine)-*block*-Poly(ethylene oxide) Diblock Copolymers. Macromol. Chem. Phys..

[41] Zheng P., Jiang X., Zhang X., Zhang W., Shi L. (2006). Formation of Gold@Polymer Core−Shell Particles and Gold Particle Clusters on a Template of Thermoresponsive and pH-Responsive Coordination Triblock Copolymer. Langmuir.

[42] Azzam T., Eisenberg A. (2007). Monolayer-Protected Gold Nanoparticles by the Self-Assembly of Micellar Poly(ethylene oxide)-b-Poly(ε-caprolactone) Block Copolymer. Langmuir.

[43] Huang D., Qi Y., Bai X., Shi L., Jia H., Zhang D., Zheng L. (2012). One-Pot Synthesis of Dendritic Gold Nanostructures in Aqueous Solutions of Quaternary Ammonium Cationic Surfactants: Effects of the Head Group and Hydrocarbon Chain Length. ACS Appl. Mater. Interfaces.

[44] Jordan V.C. (2006). Tamoxifen (ICI46,474) as a targeted therapy to treat and prevent breast cancer. Br. J. Pharmacol..

[45] Van Butsele K., Morille M., Passirani C., Legras P., Benoit J.P., Varshney S.K., Jérôme R., Jérôme C. (2011). Stealth properties of poly(ethylene oxide)-based triblock copolymer micelles: A prerequisite for a pH-triggered targeting system. Acta Biomater..

[46] Li H., Diao M., Zhang S., Wang K., Xue C. (2009). Novel Polymeric Micelles of AB2 Type α-Methoxy-Poly (ethylene glycol)-b-Poly (γ-benzyl-L-glutamate) 2 Copolymers as Tamoxifen Carriers. J. Nanosci. Nanotechnol..

[47] Nicolai T., Colombani O., Chassenieux C. (2010). Dynamic polymeric micelles versus frozen nanoparticles formed by block copolymers. Soft Matter.

[48] Van Butsele K., Cajot S., Van Vlierberghe S., Dubruel P., Passirani C., Benoit J.-P., Jerome R., Jerome C. (2009). pH-Responsive Flower-Type Micelles Formed by a Biotinylated Poly(2-vinylpyridine)-*block*-poly(ethylene oxide)-*block*-poly(*ε*-caprolactone) Triblock Copolymer. Adv. Funct. Mater..

[49] Bronstein L.M., Sidorov S.N., Valetsky P.M., Hartmann J., Cölfen H., Antonietti M. (1999). Induced Micellization by Interaction of Poly(2-vinylpyridine)-block-poly(ethylene oxide) with Metal Compounds. Micelle Characteristics and Metal Nanoparticle Formation. Langmuir.

[50] Antonietti M., Wenz E., Bronstein L., Seregina M. (1995). Synthesis and characterization of noble metal colloids in block copolymer micelles. Adv. Mater..

[51] Newman J.D.S., Blanchard G.J. (2006). Formation of Gold Nanoparticles Using Amine Reducing Agents. Langmuir.

[52] Sidorov S.N., Bronstein L.M., Kabachii Y.A., Valetsky P.M., Lim Soo P., Maysinger D., Eisenberg A. (2004). Influence of Metalation on the Morphologies of Poly(ethylene oxide)-block-poly(4-vinylpyridine) Block Copolymer Micelles. Langmuir.

[53] Johnson P.B., Christy R.W. (1972). Optical Constants of the Noble Metals. Phys. Rev. B.

[54] Templeton A.C., Pietron J.J., Murray R.W., Mulvaney P.J. (2000). Solvent Refractive Index and Core Charge Influences on the Surface Plasmon Absorbance of Alkanethiolate Monolayer-Protected Gold Clusters. Phys. Chem. B.

[55] Underwood S., Mulvaney P. (1994). Effect of the Solution Refractive Index on the Color of Gold Colloids. Langmuir.

[56] Chawla J.S., Amiji M.M. (2003). Cellular uptake and concentrations of tamoxifen upon administration in poly(ε-caprolactone) nanoparticles. AAPS PharmSci..

[57] Ambrose E.J., Jame A.M., Hovich J.H.R. (1956). Differences between the electrical charge carried by normal and homologous tumour cells. Nature.

[58] Kim C.K., Ghosh P., Pagliuca C., Zhu Z.-J., Menichetti S., Rotello V.M. (2009). Entrapment of hydrophobic drugs in nanoparticle monolayers with efficient release into cancer cells. J. Am. Chem. Soc..

[59] Shuai X., Merdan T., Schaper A.K., Xi F., Kissel T. (2004). Core-cross-linked polymeric micelles as paclitaxel carriers. Bioconjug. Chem.

[60] Shuai X., Ai H., Nasongkla N., Kim S., Gao J. (2004). Micellar carriers based on block copolymers of poly (ε-caprolactone) and poly (ethylene glycol) for doxorubicin delivery. J. Control. Release.

[61] Cavallaro G., Maniscalco L., Licciardi M., Giammona G. (2004). Tamoxifen-loaded polymeric micelles: Preparation, physico-chemical characterization and in vitro evaluation studies. Macromol. Biosci..

[62] Qin J., Jo Y.S., Ihm J.E., Kim D.K., Muhammed M. (2005). Thermosensitive Nanospheres with a Gold Layer Revealed as Low-Cytotoxic Drug Vehicles. Langmuir.

[63] Chawla J.S., Amiji M.M. (2002). Biodegradable poly (ε-caprolactone) nanoparticles for tumor-targeted delivery of tamoxifen. Int. J. Pharm..

[64] Licciardi M., Giammona G., Du J., Armes S.P., Tang Y., Lewis A.L. (2006). New folate-functionalized biocompatible block copolymer micelles as potential anti-cancer drug delivery systems. Polymer.

[65] Asadishad B., Vosoughi M., Alamzadeh I., Tavakoli A. (2010). Synthesis of folate-modified, polyethylene glycol-functionalized gold nanoparticles for targeted drug delivery. J. Dispers. Sci. Technol..

[66] Mahmoudi M., Simchi A., Imani M., Hafeli U.O. (2009). Superparamagnetic Iron Oxide Nanoparticles with Rigid Cross-linked Polyethylene Glycol Fumarate Coating for Application in Imaging and Drug Delivery. J. Phys. Chem. C.

[67] Amaral A.J.R., Emamzadeh M., Pasparakis G. (2018). Transiently malleable multi-healable hydrogel nanocomposites based on responsive boronic acid copolymers. Polym. Chem..

